# Predictors of nursing home admission of individuals without a dementia diagnosis before admission - results from the Leipzig Longitudinal Study of the Aged (LEILA 75+)

**DOI:** 10.1186/1472-6963-10-186

**Published:** 2010-06-29

**Authors:** Melanie Luppa, Tobias Luck, Herbert Matschinger, Hans-Helmut König, Steffi G Riedel-Heller

**Affiliations:** 1 Department of Psychiatry and Psychotherapy, Public Health Research Unit, University of Leipzig, (Semmelweisstraße 10), Leipzig, (04103), Germany; 2 Department of Social Medicine, University of Leipzig, (Philipp-Rosenthal-Straße 55), Leipzig, (04103), Germany; 3 LIFE Center, University of Leipzig, (Philipp-Rosenthal-Straße 27), Leipzig, (04103), Germany; 4 Department of Psychiatry and Psychotherapy, University of Leipzig, (Semmelweisstraße 10), Leipzig, (04103), Germany; 5 University Medical Center Hamburg-Eppendorf, Department of Medical Sociology and Health Economics, (Martinistraße 52), Hamburg, (20246), Germany

## Abstract

**Background:**

In previous decades a substantial number of community-based studies mostly including dementia cases examined predictors of nursing home admission (NHA) among elderly people. However, no one study has analysed predictors of NHA for individuals without developing dementia before NHA.

**Methods:**

Data were derived from the Leipzig Longitudinal Study of the Aged, a population-based study of individuals aged 75 years and older. 1,024 dementia-free older adults were interviewed six times on average every 1.4 years. Socio-demographic, clinical, and psychometric variables were obtained. Kaplan-Meier estimates were used to determine mean time to NHA. Cox proportional hazards regression was used to examine predictors of long-term NHA.

**Results:**

Of the overall sample, 7.8 percent of the non-demented elderly (n = 59) were admitted to nursing home (NH) during the study period. The mean time to NHA in the dementia-free sample was 7.6 years. Characteristics associated with a shorter time to NHA were increased age, living alone, functional and cognitive impairment, major depression, stroke, myocardial infarction, a low number of specialist visits and paid home helper use.

**Conclusions:**

Severe physical or psychiatric diseases and living alone have a significant effect on NHA for dementia-free individuals. The findings offer potentialities of secondary prevention to avoid or delay NHA for these elderly individuals. Further investigation of predictors of institutionalization is warranted to advance understanding of the process leading to NHA for this important group.

## Background

Demographic changes will lead to a considerable increase in the numbers and proportion of elderly in most developed countries after the year 2010 [1]. This demographic trend associated with a higher incidence of chronic conditions and a rapid advance in medical technology may cause a steep rise in the number of institutionalised elderly people [1]. In Germany, the number of nursing home residents increased by nearly 6 percent between the years 2003 and 2005, and by 18 percent since 1999. Altogether, 32 percent of individuals in need of care live in nursing homes [2].

The research looking at risk factors of NHA has substantially increased in the last decades [3-5]. Many early studies analysed factors using a cross-sectional design and/or univariate analyses, respectively [6,7]. However, cross-sectional studies provide no information about the future risk of NHA, and univariate analyses prevent investigators from checking for confounding effects of other variables. Previous *longitudinal *research examining predictors of NHA in old age with *multivariate *analyses has been conducted in population-based community samples [5] and in samples limited to individuals with dementia [4]. Many former community-based studies included dementia itself as baseline predictor, and found that dementia is a strong and consistent predictor and a frequent reason for nursing home admission [5]. To analyze predictors of NHA for individuals without dementia, a few other studies examined samples excluding individuals with a dementia diagnosis at baseline [8-10], not taking into consideration that some individuals develop dementia over the course of the study causing subsequent NHA. Until now, no study analysed predictors of NHA for the comparatively small but yet important group of older adults, not developing dementia before NHA. The aim of our study was to investigate predictors of NHA of a population-based sample of adults aged 75 years and older without developing dementia before NHA.

### Conceptual framework

For structuring the predictor variables of the study, we rely on the behavioural model of health service use developed by Andersen [11,12] as the conceptual framework. This model suggests that people's use of health services, or NHA, is a function of their *predisposition *to use services, factors which *enable *or impede use, and their *need *for care. *Predisposing *variables were demographic factors, social characteristics, and health beliefs. They represent the sociocultural element of the behavioural model. The *enabling variables *contain factors which make health services and NHA available and include both personal/familial and community resources. First, people must have the means and knowledge to get to those services and make use of them. Second, health personnel and facilities must be available for individuals. The *need component *is specified as the most immediate cause of health service use, and involves both perceived and evaluated health status. *Perceived need *included the amount of illness that an individual perceives and explains individuals care-seeking and adherence to medical regimens, while *evaluated need *is more closely related to the kind and amount of treatment to be provided.

## Methods

### Subjects

Data were derived from the Leipzig Longitudinal Study of the Aged (LEILA 75+), a populations-based study on the epidemiology of dementia and mild cognitive impairment. A total of 1,500 community-dwelling individuals aged 75 years and over (75-99, mean = 81.5) and residents in Leipzig, Germany, were identified by systematic random sampling from an age-ordered list provided by the local registry office. Institutionalised individuals were included in the study on a proportional basis (n = 192). The study design of the LEILA 75+ has been described in detail elsewhere [13].

**Figure 1:**
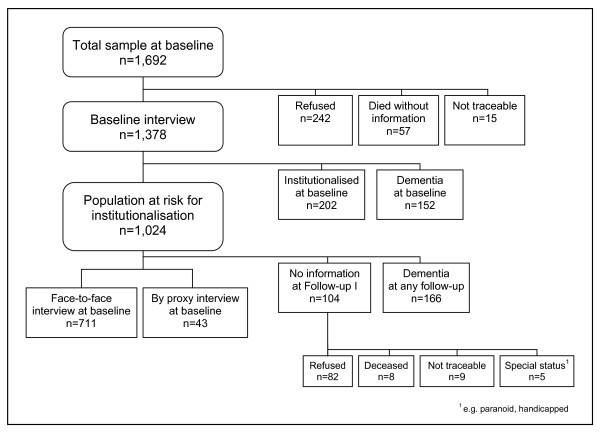
**Sampling flowchart of the study**.

Of the overall sample of 1,692 subjects, 1,265 (74.8%) individuals were interviewed face-to-face at baseline, and for 113 study participants, a fully structured proxy interview with their relatives or caregivers was conducted. Therefore, information on 1,378 (81.4%) was gathered during the baseline wave between January 1997 and June 1998. Of the 1,378 subjects available for follow-up, 202 were already institutionalised at baseline, and 152 further individuals were diagnosed with dementia according to DSM-IV criteria [14]. As a result, 1,024 participants constitute the dementia-free population at risk (figure 1). These participants were requested to take part in up to five follow-up assessments conducted between July 1998 and April 2005, on average every 1.4 years.

### Measures

Structured clinical interviews were conducted by trained physicians and psychologists during visits to the participants' homes. In addition, structured third party interviews were conducted in order to obtain information on cognitive and psycho-social functioning and subjective memory impairment.

#### Independent variables

As *predisposing variables*, we obtained socio-demographic and social structure characteristics, including age, gender, marital status, living situation, as well as education as *enabling personal factors*. As a variable for *perceived need *for care, the *subjective health status *at baseline was examined, with the question: "How would you rate your current health status?" using a 5-point Likert scale ranging from "very bad" to "very good". For the *evaluated need *for care, the following health conditions were examined: *stroke, myocardial infarction and diabetes *(coded as yes or no). *Major Depression *was assessed by means of the Structured Clinical Interview for DSM-III-R (SCID; [15]).

The Structured Interview for Diagnosis of Dementia of Alzheimer Type, Multi-infarct Dementia and Dementia of other Aetiology according to ICD-10, DSM-III-R and DSM-IV (SIDAM; [16]) was used to identify individuals developing dementia. The SIDAM comprises a test performance part and a section of clinical judgement and third-party information. The SIDAM test performance part consists of a range of cognitive tests which constitute a short neuropsychological battery with 55 questions including all items of the Mini-Mental State Examination (MMSE; [17]) which were used to measure *cognitive function*.

The capacity to perform activities of daily living (ADL/IADL) was assessed with the ADL/IADL scale according to Schneekloth et al. [18,19]. The latter scale consists of 26 items and has been developed according to an internationally used ADL/IADL list [20]. Study participants rated their ability to carry out ADL (e.g. dressing, bathing, using the toilet, stairs climbing) and IADL (meal preparation, housekeeping, shopping, use the telephone, taking their medications) with or without aid on a 3-point scale ranging from "able to do without difficulty" to "not able at all".

As further *need characteristics *we included the number of GP visits, specialist visits as well as hospital use in the prior 12 months, and the use of home help at baseline.

#### Dependent variable

Long-term NHA was defined as entry into an old-age home or nursing home at any time during follow-ups and stay until the end of the study or until death. Typically, the change in residence was ascertained when a participant was contacted for follow-up assessment. For participants who died between two waves, fully structured proxy interview with their relatives were conducted, and the time of NHA was assessed.

### Statistical analyses

Differences in socio-demographic characteristics between institutionalised and non-institutionalised individuals were investigated using two-sided t-test and χ^2^-analysis.

Time until NHA was measured in days from the date of the baseline assessment to either the date of admission to an institution, the date of death (without prior NHA), or the date of the last contact. Subjects who died at home as well as subjects still at home after 7 years or who were lost to follow-up were treated as censored observations.

Kaplan-Meier estimates were used to determine the time until NHA. The Log-rank test was used to compare the survival probabilities of different groups. To calculate the relative risk of institutionalisation, hazard ratios (HRs) and 95% confidence intervals (95% CIs) were obtained by Cox proportional hazard models. 'Enter' (all model variables are entered in one step) and 'forward stepwise' (significant model variables are entered sequentially beginning with the variable with the highest significant score) methods were used for multivariate analyses. In addition, Schoenfeld residuals were calculated in order to test the proportional hazards assumption of the Cox proportional hazards model. The statistical analyses were performed using SPSS Statistical Software for Windows, version 15.0 and STATA statistical software, release 10.0. The level of statistical significance was set at 0.05 for all analyzes.

### Ethical approval

The ethics committees of the University of Leipzig approved the study. Written informed consent was obtained from all participants.

## Results

Of the 1,024 subjects representing the population at risk for NHA at follow-up waves, 270 individuals were excluded: 166 individuals were diagnosed with dementia according to DSM-IV criteria [14] over the course of the study, and for 104 individuals only baseline information was gathered. Thus, a total of 711 (69.7%) dementia-free participants were examined face-to-face at baseline and at least at follow-up 1, and further 43 were examined by proxy (figure 1). Therefore, information on 754 (73.6%) was gathered for baseline and at least follow-up 1. Of the 754 individuals, not diagnosed with dementia over the course of the study, 59 were admitted to NH during the five follow-up waves; 15 at follow-up 1, 10 at follow-up 2, 14 at follow-up 3, 12 at follow-up 4, and 8 at follow-up 5.

The baseline characteristics of the 59 institutionalised and the 695 non-institutionalised individuals are shown in table 1. Institutionalised individuals were older (83.7 vs. 80.6 years, T = -3.94, p < 0.001) and more often widowed (68% vs. 50%, χ^2 ^= 9.37, p < 0.025) than non-institutionalised. They did not significantly differ from non-institutionalised individuals in cognitive status (MMSE-Score; 26.9 vs. 27.4, T = 1.84, p = 0.06).

**Table 1:** Sample characteristics at baseline

Characteristics	Non-institutionalised (n = 695)	Institutionalised (n = 59)	Test statistic	p-value
Age, mean (s.d.)	80.6 (4.4)	83.7 (5.9)	T=-3.94	<0.001

Gender, n (%)				
Male	204 (29.4)	15 (25.4)	χ^2 ^= 0.41	0.523
Female	491 (70.6)	44 (74.6)		

Education, n (%)				
Low	131 (18.8)	13 (22.0)	χ^2 ^= 0.82	0.665
Middle	453 (65.2)	35 (59.3)		
High	111 (16.0)	11 (18.7)		

Marital status, n (%)				
Single	57 (8.2)	5 (8.5)	χ^2 ^= 9.37	0.025
Married	235 (33.8)	9 (15.3)		
Divorced	59 (8.5)	5 (8.5)		
Widowed	344 (49.5)	40 (67.8)		

Living situation, n (%)				
Alone	411 (59.1)	48 (81.4)	χ^2 ^= 10.73	0.005
With spouse	228 (32.8)	9 (15.2)		
With others	56 (8.1)	2 (3.4)		

MMSE-Score, mean (s.d.)	27.4 (2.0)	26.9 (2.1)	T = 1.84	0.061

MMSE - Mini Mental State Examination, s.d. - standard deviation

**Figure 2:**
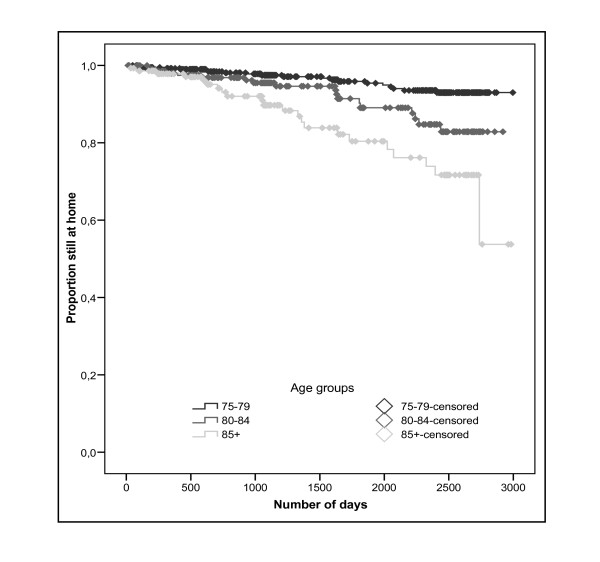
**Kaplan-Meier survival curves of the time until nursing home admission in dementia-free individuals by age groups**.

The mean time until NHA in the dementia-free sample was 2,792 days (95% CI 2,742-2,842) or 7.6 years (95% CI 7.5-7.8). Figure 2 presents the Kaplan Meier survival curve for three age groups with a significant decrease of time for increased age (Log rank: χ^2 ^= 28.37, p < 0.001). The mean time for age 75 to 79 was 2,885 days or 7.9 years (95% CI 2,835-2,934), for age 80 to 84 2,689 days or 7.4 years (95% CI 2,589-2,790) and for individuals aged 85 and over 2,487 days or 6.8 years (95% CI 2,306-2,669).

In table 2 the results of the Cox proportional hazards models are shown. First, a multivariate Cox regression model was performed in order to analyze the effect of all included variables ('Enter' method). The baseline variables found to be significantly associated with a shorter time to institutionalisation were increased age, living alone (compared to those living with someone besides a spouse), a very low self-rated health status (compared to satisfying or good health status), functional and cognitive impairment (without dementia), major depression, stroke, myocardial infarction, and a low number of specialist visits. Gender, marital status, education, diabetes, number of GP visits, prior hospital use, and paid home help use had no significant effect on time until NHA. The model chi-square difference of 81.58 was significant at the <0.001 level. The proportional hazards assumption was met for all included variables (χ^2 ^= 31.18, p = 0.12). Second, we performed a multivariate Cox regression model, entering significant variables sequentially, with checking and possible removing variables that became non-significant ('forward stepwise' method). In this model, the remaining baseline variables associated with a shorter time until NHA were increased age, living alone (compared to those living with spouse or with others), functional and cognitive impairment, major depression, stroke, myocardial infarction, low number of specialist visits and paid home help use. The model chi-square difference of 73.23 was significant at the <0.001 level. The proportional hazards assumption was met for all included variables (χ^2 ^= 13.68, p = 0.19).

**Table 2:** Predictors of Nursing Home Admission - Cox proportional hazards model ('enter' and 'forward stepwise' model)

	All variables in the model ('enter' method)			Significant variables sequentially entered in the model ('forward stepwise' method)		
**Predictors**	**HR**	**CI 95%**	**p value**	**HR**	**CI 95%**	**p value**

Age at baseline	1.10	1.03-1.16	**0.003**	1.09	1.03-1.15	**0.005**

Gender	0.73	0.31-1.74	0.476	-	-	-

Marital status (ref. = single)						
married	0.58	0.05-6.19	0.648	-	-	-
divorced	0.78	0.20-3.02	0.713			
widowed	0.74	0.28-2.01	0.559			

Living situation (ref. = alone)						
with spouse only	0.32	0.04-2.75	0.297	0.31	0.13-0.72	**0.006**
with others	0.11	0.01-0.85	**0.034**	0.11	0.01-0.88	**0.037**

Education (ref. = low)						
middle	1.33	0.60-2.94	0.480	-	-	-
high	1.73	0.57-5.23	0.334			

Self-rated health status (ref. = very low)						
satisfying	0.19	0.04-0.89	**0.035**	-	-	-
good	0.14	0.03-0.69	**0.016**			

Functional impairment (ADL/IADL score)	3.01	1.12-8.13	**0.030**	3.08	1.28-7.44	**0.012**

Cognitive Impairment (MMSE Score)	0.81	0.69-0.94	**0.006**	0.84	0.74-0.97	**0.015**

Major Depression	9.91	2.02-48.55	**0.005**	8.68	1.91-39.52	**0.005**

Stroke	4.86	2.15-10.98	**0.000**	4.77	2.13-10.69	**0.000**

Myocardial infarction	2.07	1.14-3.77	**0.017**	2.20	1.29-3.77	**0.004**

Diabetes	0.68	0.30-1.57	0.370	-	-	-

Number of GP visits	0.95	0.63-1.42	0.789	-	-	-

Number of specialists visits	0.61	0.44-0.85	**0.003**	0.64	0.48-0.87	**0.004**

Prior hospital use	1.08	0.71-1.66	0.708	-	-	-

Prior paid home help use	2.19	0.91-5.27	0.079	2.42	1.05-5.58	**0.038**

## Discussion

The aim of the study was to analyze predictors of long-term NHA in a population-based sample of individuals aged 75 years and older who did not develop dementia before their nursing home admission.

We found that increased age, living alone as *predisposing variables*, and functional and cognitive impairment, major depression, 12 months-history of stroke and myocardial infarction, and a low number of specialist visits as *need variables *lead to an increased risk of NHA for dementia-free individuals. A comparison of our results with findings of community-based studies summarized in a recent review of Luppa et al. [5] revealed that especially, age, living situation, functional and cognitive impairment were also strong predictors for NHA in representative samples of community-dwellings. In contrast to our findings, rather inconsistent results were shown for depression, stroke and heart disease in the previous literature (significant as well as non-significant effects) for community-based studies [5]. However, these findings may be attributed to the methodical difference that studies included in this review did not consequently exclude dementia cases from the samples.

In comparison with *numerical *results of these community-based studies (mostly with inclusion of dementia cases) [5], the effect of increased age and cognitive impairment was lower for our dementia-free sample as summarized for community-based studies (HRs for age: 1.09 vs. 1.06-7.72, and cognitive impairment: 1.19 vs. 1.59-1.67). In contrast, for living alone, functional impairment, myocardial infarction, stroke and major depression, and the effect on institutionalisation was appreciably increased (HR for living alone: 3.23 vs. 1.72, functional impairment: 3.08 vs. 1.05-2.50, myocardial infarction: 2.20 vs. 1.36-1.47, stroke: 4.77 vs. 1.09, and major depression: 8.68 vs. 1.01-2.38). A view of those studies examining a sample dementia-free only at baseline sample showed - consistent with our results - a significant effect of age, functional impairment, and stroke, and also non-significant effects for gender and self-rated health status [8]. However, as for the above-mentioned community-based studies, higher effects for age and lower effects for functional impairment and stroke were found. McCallum et al. [9] confirmed our results of an increased risk of NHA for higher age, functional impairment and depression; however, also with a slightly higher effect for age and a lower effect of functional impairment and depression. Yet one should keep in mind that McCallum et al. [9] and Andel et al. [8] examined individuals dementia-free only at baseline. Thus, a number of individuals probably developed dementia before NHA, causing subsequent admission (see also introduction). Looking at our results in relation to previous research, it can be summarized that predictors of NHA for individuals who did not develop dementia before NHA were for the main part severe physical or psychiatric diseases such as stroke, myocardial infarction, and major depression, which were mostly associated with functional impairment. Beyond this, living alone and, to a lesser extent, higher age and cognitive impairment were further significant predictors of NHA.

Aside from that, we found that the effect of self-rated health status on NHA changed from statistically significant to non-significant from the 'enter' model to the 'forward stepwise' model. Thus, the variable was removed from the 'forward' model, probably because its effect was sufficiently explained by other rather objective need measures such as functional and cognitive impairment or existing diseases. Another meaningful finding was that with an increasing number of specialist visits, the risk of NHA decreased. In the German health care system almost all individuals are medical insured, which includes direct access to specialists without further out-of-pocket expenses. Therefore, an effect of the socioeconomic status on frequency of specialist's visits could be ruled out to a large extent; and also due to the fact that the model was adjusted by educational status. Actually, frequent visits to specialists suggest a heightened medical comorbidity mostly associated with a low functional status, known as variables leading to premature NHA. However, in multivariate analyses adjusting the effects of other independent variables, the positive effect of appropriate treatment of medical conditions by specialists on institutionalisation was detectable, and hence offers a possibility for the patients and their relatives to prevent premature NHA by seeking specialists for dealing with diseases.

In total, 7.8% of the original dementia-free sample was admitted to NH during the study period. For the individuals with an incident dementia diagnosis which we did not include in our analyses, 47.7% had become residents of a NH by the end of the study [21]. Compared to the results of current community-based studies with similar length of study, our admission rate can be considered rather low; studies with a length between nine and eleven years showed average admission rates between 10.9% and 33.8% over this period [10,22-25]. However, these admission rates were conclusively substantiated by the inclusion of dementia cases with marked shortened time to NHA [5].

### Limitations

In view of previous research, many further variables not requested in the interviews, and thus not included in the analyses of the present study, affected the risk of NHA. Especially for a dementia-free sample, such factors with inconsistent outcomes in community-based studies like social support, income or disorders such as hip fracture and incontinence (cf. [5]) were of great interest and may be addressed in future research.

## Conclusion

Most elderly people prefer to remain in their homes, because they can keep their social network, preserve environmental landmarks, and enjoy a better quality of life. The findings of our study identified predictors of NHA of dementia-free older adults, and therefore offer potentialities of secondary prevention to avoid or delay NHA for these elderly individuals. Practical implications include the identification and treatment of older adults at high risk for developing severe physical or psychiatric diseases such as stroke, myocardial infarction or major depression. Furthermore, consequent rehabilitation of older adults who have had a myocardial infarction or stroke in order to prevent another cardiovascular event should be targeted. Moreover, there should be a strong focus on disabled elderly people living alone to achieve support by home- and community-based services. Implications for further research should include in-depth investigations of the complex mechanisms leading to NHP of individuals without a dementia diagnosis. The individual affliction, the socioeconomic consequences, and the distress associated with nursing home admission require further systematic, prospective investigations of predictors of institutionalization to advance understanding of the process leading to nursing home admission. Qualitative research might be also a helpful tool to fully elucidate the decision process and critical turning point for possible interventions for this important subgroup.

## Competing interests

The authors declare that they have no competing interests.

## Authors' contributions

ML, HM, SRH, and HHK designed the study and formulated the research questions. ML conducted the literature search and wrote the manuscript. ML, TL, HM analysed and interpreted the data. SRH and HHK revised the manuscript. All authors read and approved the manuscript.

## Pre-publication history

The pre-publication history for this paper can be accessed here:

http://www.biomedcentral.com/1472-6963/10/186/prepub
